# Social and Emotional Competences in Spain: A Comparative Evaluation Between Spanish Needs and an International Framework Based on the Experiences of Researchers, Teachers, and Policymakers

**DOI:** 10.3389/fpsyg.2019.02127

**Published:** 2019-09-20

**Authors:** Pilar Aguilar, Isabel Lopez-Cobo, Francisco Cuadrado, Isabel Benítez

**Affiliations:** ^1^Department of Psychology, Universidad Loyola Andalucía, Seville, Spain; ^2^Department of Communication and Education, Universidad Loyola Andalucía, Seville, Spain

**Keywords:** social and emotional competences, learning to be, Spanish schools, international toolkit, educational policies

## Abstract

Critical aspects in the field of education are currently related to low levels of socioemotional competences and high rates of school dropouts. However, there are no standard practices or guidelines for helping countries to assess and train social and emotional competences. To overcome this limitation, the project *Learning to be* (L2B) aims to propose a comprehensive model of the assessment and development of social and emotional competences that bring together policymakers, researchers, teachers, school authorities and learners from different participating countries: Finland, Italy, Latvia, Lithuania, Portugal, Slovenia, and Spain. The project attempts to create an international framework for regulating activities and supporting teachers working on the social and emotional competences of the students and to provide tools which can be used across countries. The aim of the paper is to describe how L2B has been developed in Spain by analyzing the contributions of the international framework and extracting the specific needs in the Spanish context. Specifically, we describe two products of the project: (a) the process for creating a *toolkit*, a tool focused on creating activities and materials for helping teachers develop social and emotional competences of students and (b) the results of an exploratory study in Spain in which social and emotional competences are assessed and compared to the international framework. First, we explain the definition of social and emotional competences used for creating the *toolkit*. Second, we analyze responses of Spanish students to questionnaires administered before applying the *toolkit* in the schools. The paper describes social and emotional competences in Spain and evaluates the overlap between relevant variables in Spain and those used for developing the *toolkit*, the international tool. Specific needs in Spain are discussed, as well as the contribution of the project for promoting the improved development of students’ social and emotional competences, improving learning outcomes and stronger social cohesion in schools and local communities, as well as the utility of the results for creating new educational policies.

## Introduction

The construction of safe and effective educational environments is achieved through holistic contributions that improve the effectiveness of social and emotional learning (SEL) initiatives. As previous studies have shown, the importance of SEL lies on the impact it has on relevant issues such as youth development. For instance, Taylor et al. (2017) conducted a meta-analysis of follow-up effects of SEL programs and found that students who participated in school-based SEL, improved skills, positive attitudes, prosocial behavior, academic performance and increased their wellbeing. Furthermore, these authors confirmed that positive effects maintained after months of participation in the program.

According to the study by Catalano et al. (2012), different prevention programs developed over the last three decades and based on SEL showed a great degree of efficacy at reducing adolescent problems behavior. These authors conclude that most of the programs have focused the intervention in three different areas. (1) Family risk factors including parenting skills, parent-child communication, and affective relationships (e.g., Strengthening Families Programs for Parents and Youth 10–14, New Beginnings Programs or Functional Family Therapy); (2) school environment, working on reducing aggression, crime, alcohol, tobacco use, unwanted pregnancies, sexually transmitted diseases and mental health symptoms and disorders (e.g., Seattle Social Development Project, Gatehouse Project); and (3) peer risk factors, with benefits in reducing alcohol and other substance, delinquency, risky sexual activity, academic failure or in increasing psychosocial competencies (e.g., Unplugged and Life Skills Training, Positive Adolescent Training Through Holistic Social Programs or Stepping Stones). As these authors conclude, “employing a combination of programs and policies that engage schools, families, and communities will probably yield long-term beneficial effects” (Catalano et al., 2012, p. 6).

The teaching staff is, thus, a key element for the promotion and dissemination of practices at both classroom and school levels, by coordinating actions focused on developing children in their own environments, such as their community or families. This idea implies that social and emotional skills must be exercised in the same way as other skills such as reading, writing or mathematical thinking.

The Collaborative for Academic, Social and Emotional Learning (CASEL) identifies a list of closely related competences that should be considered in school programs focused on children’s holistic development. These competences are those related to cognitive, affective and behavioral fields. CASEL groups them into five key domains (Casel., 2015):

(a) Self-awareness: This domain allows recognizing one’s own emotions, strengths and self-efficacy elements, while, at the same time, favoring an adequate self-perception and confidence in one’s own abilities.
(b) Self-management: This domain is focused on the control of impulse and stress through the establishment of objectives that favor self-discipline, self-motivation and the development of organizational skills.
(c) Social awareness: This domain focuses on the ability to develop empathy involves taking perspective, appreciating diversity and respecting others and their differences.
(d) Social management: This domain focuses on establishing and maintaining positive relationships, developing active listening and empathic communication, and seeking and offering help if necessary, which are all necessary skills to effectively develop the group work so important in modern society.
(e) Responsible decision-making: This domain implies that the student must go beyond identifying the problem, analyzing and evaluating the situation or solving the problem; this competence also involves doing everything with a social conscience and acting with ethical responsibility.

These competences can be developed and trained in all areas of student life. This fact is the reason why students largely internalize competences when strategies, practices and policies of SEL are intentionally developed throughout these contexts. Systemic SEL, according to CASEL, involves a coordinated strategy across classrooms, schools, households, and communities. The degree of quality in the relationships and interactions between these areas determines the success of SEL development.

In response to the scope of the classroom, CASEL found that the interactions between peers and the teacher are presented as a fundamental part of students’ social and emotional development. In this way, a relationship characterized by support and trust leads to an improvement in social competence, positive active commitment and academic improvement. Furthermore, students extend beyond the classroom itself, learning in informal environments in the school. It is therefore necessary to establish a culture of care at school, encouraging ethical values and expectations related to social relationships and behavior, and promoting respect and kindness toward others; this is the relevant role that educational leadership plays. Previous research also indicates that those schools where the educational leadership prioritizes SEL obtain successful implementation (Devaney et al., 2006).

In addition, the family school partnership is crucial for the successful development of SEL. Those programs in which both families and schools are focused on authentic relationships and based on active participation, listening and respect, are those that present the most effective programs (Albright and Weissberg, 2010). Likewise, this positive school–home relationship promotes students’ higher participation rate in the classroom, as well as lower dropout rates (Greenberg et al., 2003).

The last key element discussed by CASEL (2015) is the importance of establishing strong relationships between community partners and organizations that unify efforts and contribute to the development of SEL through the generation of educational initiatives that give students the opportunity to apply acquired skills (Durlak et al., 2010; Oberle et al., 2016). In that sense, many countries work on these through extracurricular activities related to sports, art or religion.

In terms of effective SEL practices and their integration in the school curriculum, previous reviews and meta-analyses (Durlak et al., 2011; Corcoran et al., 2018; Yang et al., 2019) highlight a list of indicators that are needed for successful SEL initiatives. On one hand, these indicators refer to the characteristics of the programs, affirming that they should be based on rigorous scientific theories and implemented by highly trained staff members who can monitor the development of these initiatives. In that sense, it is recommended to measure the quality of the implementation and to analyze the sustainability of the programs over time; in this line, school leaders and administrators have to consider SEL as a core part of the educational mission. On the other hand, the authors identified elements related to the curriculum, emphasizing that SEL can be integrated into the curriculum with other core subjects. In that way, teachers can apply a formative assessment which reduces comparisons among students, thus eliminating the competitiveness which embedded in daily interactions among the educational community; it would also create caring classrooms and more localized schoolwide environments. Other indicators highlighted in the meta-analyses are those related to the students’ context. The importance to interconnect different contexts in which the students develop is noticed, so these authors’ strategies extend beyond the school or the particular educational stage of interest.

Common options for developing SEL are discussed. For instance, Zins and Elias (2007) explain that SEL can range from the smallest to the most substantial changes in school life. There are different forms of SEL development in the school and some of them include a specific curriculum focused on learning SEL. Other approaches go through the development of interconnected activities that ensure simultaneous training along with the curricular subjects. Last, there are schools adopting the integration of SEL in extracurricular activities, which is the most commonly mentioned option in Elliott and Mihalic (2004). These authors indicate that using only extracurricular activities seems to have limited effects, because of the limited nature of that environment. In most cases, the absence of national guidelines means that schools individually promote SEL by using diverse strategies according to their own interests and motivations.

What previous research undoubtedly underlines is that strategies habitually adopt partial and segmented implementation of SEL, without temporal continuity or an evaluation of the results, thus leading to ineffective outcomes for students (Cahill et al., 2019). Consequently, and according to previous experiences applying SEL programs in schools, the need to create activities focused on connecting different areas and contexts of children’s daily lives is clear. As scientific literature confirms (Birch and Ladd, 1997; Zins et al., 2004; Rimm-Kaufman and Hamre, 2010), SEL is closely related to students’ academic achievement and offers an additional value for schools to make efforts on incorporating SEL in an optimal and successful way throughout all educational stages and in the curriculum.

Finally, looking at trends in the development of educational policies from a global perspective, training on SEL promotes economic growth and social cohesion in Europe. The individuals with a balanced set of cognitive as well as social, emotional and health (SEH) skills are more likely to cope with difficult situations in life, perform well in the labor market, and achieve personal and professional goals (OECD, 2015).

In the specific case of Spain, The Organic Law 2/2006 of Education (LOE) made special emphasis on social and emotional education and learning. The preamble itself states that education is the most appropriate way to build students’ personality and “to help them develop their abilities to the maximum, to shape their own personal identity and to contribute to their understanding of reality, integrating the cognitive, affective and axiological dimension.” Thus, the aim of education is to ensure that all citizens “achieve the maximum development of all their capacities: individual and social, intellectual, cultural and emotional, for what they need to receive a quality education adapted to their needs.” Specifically, article 71 states that “educational administrations shall provide the necessary means for all students to achieve maximum personal, intellectual, social and emotional development, as well as the objectives established in general terms in this Law.”

The educational system in Spain is decentralized, so the educational authorities (together with health and social affairs) are transferred to autonomous administrations. These autonomous governments have a certain degree of autonomy for concretizing and extending the national policies. In this sense, and in the case of social and emotional learning, each autonomous community has marked its own statements, within the margins allowed by central administrations.

In this context, different programs have been launched for the development of emotional and social skills. On one side, the development of emotional intelligence is occurring more as individual and specific initiatives than as global approaches or at the level of the autonomous administrations. Apart from this, some initiatives are also aimed to provide a further development of social and emotional skills. As an example, in Cantabria, the Marcelino Botín Foundation has conducted the *Responsible Education* program, which has been already implemented in more than 100 educational centers in different parts of Spain and has shown improvements in academic performance, as well as in a reduction of students’ anxiety levels. This initiative consists of different programs specific to social and emotional development, i.e., *Reading and emotions*, *Choir of emotions*, and *Literature, Emotions and Creativity*. In Catalonia, the GROP research team (Grop de Recerca en Orientació Psicopedagògica) focuses both its research and training activities on emotional education at different levels of education, as well as for teachers’ learning (Bisquerra, 2016). Finally, the program *RULER*, which has been empirically supported in the United States, is also being applied in Spanish schools. Table 1 briefly describes characteristics of the validated programs applied in Spain, as well as their main results.

**TABLE 1:** Validated programs carried out in the Spanish context.

**Program**	**Population**	**Skills**	**Results**	**Measures**
APRENDE A CONVIVIR/Learn to live together	3–7 years old	Rules Feelings Emotions Empathy Communication skills Interpersonal skills Problem solving skills	Prosocial attitudes predicted positively and significantly, students’ academic performance, Perceived Emotional Intelligence (PEI)	Three evaluation instruments created by the authors Acquisition of the program content Direct evaluation with students and Indirect evaluation by teachers Dynamics of the teaching/learning process
INTEMO (Emotional Intelligence in teenagers)	12–18 years old	Perception and expression Emotional facilitation Emotional understanding Regulation	The program decreases physical and verbal aggression levels, anger, hostility, personal discomfort. It increases empathic abilities, especially in boys It improves clinical symptoms and results are maintained after 6 months later	Spanish adaptation of Behavior Assessment System for Children and adolescents (BASC)
EDUCACIÓN EMOCIONAL/Emotional Education (GROP)	6–12 years	Emotional awareness Emotional regulation Autonomy Life and well-being skills	Improvement in all dimensions	
EDI Program	10–11 years	Interpersonal skills Managing emotions Adaptability General mood	Improvement in all dimensions	Spanish version of the Emotional Quotient Inventory (EQ-i:YV)
Programa curricular socioemocional (SOE)	Primary Education	Prosocial behavior and sociability Assertiveness Emotional regulation and management Interpersonal conflicts resolution	Satisfaction of teachers involved	Sociometric questionnaire of peer nomination
Happy 8–12 Gamified program of Emotional Education	Primary Education		Significantly improves emotional competences, social climate in classroom and playground (fewer number of conflicts between peers). Decreases anxiety levels, Increases significantly academic performance	Emotional Development Questionnaire for Children (CDE)
Primary School Teachers (Barcelona and Lleida) (GROP model)	Teachers	Awareness Regulation Autonomy Social skills Well-being	Improvement in emotional intelligence and social environment	
RULER (Marc Brackett)	All ages	Ability to recognize Understand, label and regulate emotions in an effective way		Utrecht Work Engagement Scale (UWES)
				Emotion-Focused Interaction Scale (EFIS)
				Maslach Burnout Inventory-Educators Survey (MBI-ES) Positive and Negative Affect Schedule (PANAS) Big Five Inventory (BFI-44)

However, there are programs being applied in Spanish schools that do not have evidence collected on their utility. Some of these programs are presented in Table 2.

**TABLE 2:** Non-validated programs carried out in the Spanish context.

**Program**	**Population**	**Skills**
Programa Aulas Felices (Happy classrooms program)	Primary Education	Personal and social development of students
		Promote happiness of children
S.I.C.L.E. (Siendo Inteligente Con Las Emociones/Being Intelligent with the emotions)	Secondary Education	Emotional skills and empathy
ULISES Program	10–12 years	Developing emotional self-control
		Prevention of drug use and abuse
RAPPORT (Emotional Communication)	Teachers	Communication skills in teachers
Botín Foundation	Students of different ages	Different SEL programs for different skills development

Despite the evolution of SEL programs in Spain, much remains to be done, especially in the rigorous evaluation of the actions and interventions; therefore, program sustainability strategies must be developed. In most cases, a timely intervention without a follow-up is proposed. In addition, it is also necessary to develop training programs for the whole educational community (not only within the classroom) in line with what is already done in other countries.

The project *Learning to be* (L2B) attempts to assemble previous experiences when conducting SEL programs, as well as unifying the approaches of professionals from different countries. Specifically, the perspectives of policymakers, researchers, teachers, school authorities and learners have been combined in order to capture needs at different levels.

L2B targets low levels of socioemotional skill development and high rates of school dropouts in Europe, factors that lead to a negative influence on the social, academic and professional lives of young people and their personal fulfillment. Because of this, one of the goals of the project is to develop an “SEL Skills Assessment Toolkit” for teachers, which could assist schools in promoting positive relationships, developing understanding, and fostering social inclusion and respect for diversity. In addition, L2B aims to overcome previous limitations due to a lack of pre- and postprogram assessments of SEL levels. With this policy measure, the project proposes to widen the set of skills to be assessed and recognized in formal and non-formal education settings for a variety of purposes, including school and university admission, career prospects, and the implementation of other social roles; this will also bring together policymakers, researchers, teachers, school authorities, and learners from seven different participant countries. To do that, some issues are pursued:

(a) A comprehensive model for assessing social, emotional, and health-related competences for schools will serve as a theoretical and methodological basis for social-emotional learning and assessment in primary and secondary schools.
(b) A practical assessment *toolkit* for educators and learners for classroom use will include (self-)assessment tools for teachers, non-formal learning, and students.
(c) An institutional self-assessment instrument for schools that will help them to evaluate their own social-emotional-health learning situation.
(d) A set of recommendations for reviewing policy on SEH skills which will initiate the analysis of educational policies and national curricula in order to better support and facilitate positive policy reforms in Europe.

To summarize, L2B is not an intervention program as previous SEL programs, L2B is a European project that aims to develop assessment methods and tools for the development of SEH -related competences in general education schools. L2B pretends to overcome limitations of previous studies in terms of evaluation by proposing formal and informal procedures for extracting evidence on the improvement of SEL between different time points. The tools will facilitate the recognition of SEH skills within the education system and would form a basis for further development of education policies and practices in the national curricula, improving the quality of SEH skills in education and fostering social cohesion and fundamental values of a democratic society.

The aim of the paper is to present two main products of the development of L2B in Spanish schools. On one hand, we will describe the *toolkit* creation process, including the description of the theoretical approaches to work on SEL dimensions and the final structure of the *toolkit*. On the other hand, we will present results from the analysis of Spanish students’ responses to questionnaires administered before using the *toolkit*. The purpose of this second part is to evaluate the needs of students in Spain and compare them to skills promoted in the *toolkit* created under international criteria.

## Phase 1: Creation of the Toolkit

The *toolkit* is the basis of the experimental intervention in the project. It provides quite simple and “down to earth” educational tools for activating these values in classrooms and school life by (a) assisting in creating a relationship-centered learning environment, (b) promoting instructional methods to support the development of social and emotional skills and (c) developing progress monitoring and assessment practices for different levels at school.

The *toolkit* creation process had the following stages:

(1) Building a workgroup among all project partners.
(2) Reporting on the current status of SEL skills assessment and development in schools in different countries.
(3) Designing an SEL skills assessment framework defining progression of skill development, indicators of progression and educational objectives for different ages of learners.
(4) Development of the *toolkit* structure based on the designed framework.
(5) Generating a draft version, revision, and final version of the toolkit.
(6) Translation and production of the toolkit into partner languages.

### Building a Workgroup Among All Project Partners

The creation of the *toolkit* was based on the experience of 25 professionals (teachers, educators, researchers, etc.) from seven participant countries. Among them were university professors, school-based educators, SEL experts from research institutes, and ministries of education authorities.

### Reporting of the Current Status of SEL Skills Assessment and Development in Schools

Each participant partner carried out current research on SEL skills assessment and development in their country’s schools. Apart from the situation in the participant countries, the search was extended to other countries outside Europe, especially in the United States. After sharing and analyzing all the different models, programs and interventions existing in the different countries as well as the indicators of success of each, CASEL’s effective SEL program was chosen as a starting point from which design and build the *toolkit*.

### Designing an SEL Skills Assessment Framework

As described in the introduction section, CASEL identifies interrelated sets of cognitive, affective and behavioral competences that school-based programs should address and that can be clustered into five key domains: self-awareness, self-management, social awareness, relationship skills, and responsible decision-making. Each domain defines a series of competences that were taken as a starting point to define the approach of the *toolkit*.

According to the CASEL model, as well as to information provided by other programs and experiences, SEL can be promoted at three levels:

- Level 1: Relationship-centered learning environments and teaching methods
- Level 2: Evidence-based SEL programs
- Level 3: Embedding SEL in the core curriculum

After reviewing the needs of the participant countries, it was decided to focus on Level 1, as it constitutes the basis for creating a safe, caring, well-managed, and relationship-centered environment (Collaborative for Academic, Social and Emotional Learning [CASEL], 2003). Moreover, that level is mandatory for implementing relevant teaching methods as well as for laying a solid foundation for the successful promotion of SEL at levels 2 and 3.

Three main goals were defined for Level 1:

- Goal 1: Self Awareness and Self-Management
- Goal 2: Social Awareness and Relationship Skills
- Goal 3: Responsible Decision-Making

For each of the goals, different SEL assessment strategies for teachers were developed, according to the standards shown in the Table 3.

**TABLE 3:** Standards to design the SEL assessment strategies based on the three goals.

**Goals**	**Standards**
Self-awareness	(1) Identify and manage one’s emotions and behavior
	(2) Recognize personal qualities and external supports
	(3) Demonstrate skills related to achieving personal and academic goals
Social awareness Relationship skills	(1) Recognize the feelings and perspectives of others
	(2) Recognize individual and group similarities and differences
	(3) Use communication and social skills to interact effectively with others
	(4) Demonstrate and ability to prevent, manage, and resolve interpersonal conflicts in constructive ways
Responsible decision-making	(1) Consider ethical, safety, and social factors in making decisions
	(2) Apply decision-making skills to deal responsibly with daily academic and social situations
	(3) Contribute to the well-being of one’s school and community

### Development of the *toolkit* Structure Based on the Designed Framework

According to previous findings, the decision taken in the exploratory stage and the definition of the framework for SEL assessment, the structure of the *toolkit* was defined as a series of chapters/sections, organized as follows:

(1) What is Social and Emotional Learning: A foundational section to define SEL, explain its benefits, present different evidence-based SEL programs and explain in detail the approach considered in the *toolkit*. This section incorporated specific subsections focused on the relationship between SEL skills and academic success as well as between SEL and mental health.
(2) Building safe and caring educational environments: This section presents SEL development across different levels of the education for children in schools (class, school, family, community), as well as the educational provisions for SEL in different types of education such as formal, non-formal and informal education.

(3) Assessment of social and emotional Skills: This chapter reveals the approach adopted to measure social and emotional skills as an ability. From this, it focuses on the practical use of the subsequent parts of the *toolkit*:

(a) SEL Standards are learning objectives for the development of social and emotional skills for different age groups of students.
(b) Formative Assessment Strategies are strategies to build a constant process of assessment and feedback in class that helps teachers and students track their progress and identify areas and needs for improvement.
(c) Instructional Tools for the Classroom use are a series of teaching techniques that support SEL in class regardless of subject or the material being learned.
(d) Students’ Self-Assessment Tools are instruments monitoring SEL progress at school on different levels including students’ learning, teachers’ practices and the schoolwide characteristics of SEL.

(4) SEL in Practice: In this chapter, a whole plan of action is presented to integrate social and emotional education into the life of the entire school as well as of the student:

(a) Planning SEL implementation in the school.
(b) Creating relationship-centered learning environments in the classroom.
(c) Teaching methods to strengthen SEL.
(d) Instructional teaching methods organized in a matrix connecting different methods, formative assessment strategies and SEL standards. According to the L2B project design and aims, all of these strategies and methods have been designed and prepared for grades 4 (ages 9 and 10) and 8 (ages 13 and 14).

(5) Tools for SEL Assessment at School: Finally, this section provides specific tools to evaluate SEL progress at the previously established levels:

(a) Students’ social and emotional skills assessment tools.
(b) Classroom assessment tools.
(c) Teachers’ self-assessment tools.
(d) School-level assessment of SEL.

The *toolkit* incorporated several annexes with different samples of the materials proposed to be used including sheets, report forms, checklists, surveys and curriculum models.

### Generating a Draft Version, Revision and Final Version of the *toolkit*

Once the final draft version of the *toolkit* was completed, it was sent for peer review by two external experts: an elementary school class teacher and an expert in SEL, particularly in the CASEL model. The review focused on the entire structure of the *toolkit*, the comprehensibility of its different sections and chapters, and the practical applicability of the *toolkit*.

Once both reviews were received, further changes were introduced in the *toolkit* to improve it according the reviewer recommendations and the final English version was finalized.

### Translation and Production of the *toolkit* Into Partner Languages

The final approved version of the English *toolkit* was translated into seven languages: Finnish, Italian, Latvian, Lithuanian, Portuguese, Slovenian, and Spanish. After translation and final editing, a proof-reading process was conducted to check both the intelligibility of the translations as well as the coherence between the different translated versions of the *toolkit*.

Once the *toolkit*, the main tool for the intervention, was ready, teachers and other professionals of the school community were trained in using the materials during daily activities in the classroom. Moreover, this training also focused on highlighting the benefits of the development of emotional and social learning programs in order to motivate the whole school community, since the success of this tool is based on the community involvement. The next step consisted of evaluating social and emotional competences as well as other relevant variables of students before starting the intervention. The main results of the evaluation are presented in the following section.

## Phase 2: Social and Emotional Needs in Spain

The aim of the second phase was to evaluate the extent to which European educational guidelines used for creating the *toolkit* are applicable to the Spanish context. Specifically, we will check whether social and emotional competences addressed by the *toolkit* respond to Spanish students’ socioemotional needs. Since the *toolkit* was intended to be a dynamic resource, the results will be used for adapting activities and materials to the Spanish context in order to improve the *toolkit.*

### Participants

The participants were 1494 primary and secondary education students (46.2% girls) aged between 7 and 16 years old (*M* = 11.45; *SD* = 2.15) from twelve schools is Spain located in the provinces of Andalucía, Madrid, Castilla y León, Galicia and Asturias. School principals in different cities were contacted and asked to participate in the project after being explained the goals and intended activities.

### Instruments

The evaluation protocol included some demographic questions as well as scales assessing social and emotional competences and personal variables such as gender, age, place of birth and family economic situation. Original versions of selected scales were adapted, when needed, to Spanish by following a back-translation process (Harkness and Schoua-Glusberg, 1998). First, instruments were translated to Spanish by a group of experts, then Spanish versions were translated to the original languages by another group of experts and both versions were compared by identifying the mistakes in the intermediate version. Data were collected between October and November 2018.

The following scales were included in the protocol:

#### The Social Emotional Competence Questionnaire (SECQ) (Zhou and Ee, 2012)

The SECQ includes the five dimensions of SEC which are self-awareness, social awareness, self-management, relationship management, and responsible decision-making. It consists of 25 items with a 6-point response format varying from *not at all true of me* to *very true of me*.

#### Short Version of the Rosenberg Self-Esteem Scale (RSES)

Self-esteem was evaluated using a short version of the Rosenberg (1979) Self-Esteem Scale. The scale included five items with statements reflecting general self-acceptance, self-respect, and overall attitude toward oneself (see Tuominen-Soini et al., 2008).

Some scales from the 2014 World Health Organization Health Behavior in School-aged Children (HBSC) survey (Currie et al., 2014) were also included:

#### Bullying

First, students were provided with a definition of bullying. Then, they were asked to respond to two questions taken from the revised Olweus Bully/Victim Questionnaire (Olweus, 1996). The questions asked about the frequency of bullying other students (perpetrator) and the being bullied (victim). Response options varied from *I have not bullied another student(s) at school during the last month/I have not been bullied at school during the last* month to *More than once a week.*

#### Life Satisfaction

This was measured by an item based on the *Cantril Ladder* technique (Cantril, 1965) which asks the student to place him or herself on a ladder (from *0* to *10*) according to his or her feelings. Response options varied from *the best possible life for you (0)* to *the worst possible life for you (10).*

#### Teacher Support

A three-item scale measures how students feel about their relationship with teachers at school. Responses ranged from 1 (*Completely disagree*) to 5 (*Completely agree*).

#### School Difficulties

Participants were asked if they faced difficulties in doing tasks related to reading and writing (taken from Finnish School Health Promotion Study, 2017). Responses ranged from 1 (*Not at all*) to 5 (*Very much*).

Variables measured through each of these scales were connected to competences described in the *toolkit*. Table 4 indicates the competences included in the *toolkit* on one side and the scales informing these competences during the evaluation on the other.

**TABLE 4:** Connection between competences trained trough the toolkit and scales administered.

**Socioemotional competences trained trough the toolkit**	**Scales**
*Self-awareness*	SECQ
*Self-management*	Self-esteem
	School difficulties
*Social awareness*	SECQ
*Relationship skills*	Teacher support
	Bullying
*Responsible decision-making*	SECQ

### Procedure

The study was conducted in accordance to the recommendations of the Spanish Organic Law 3/2018 and the Spanish Agency for Data Protection, which regulates the fundamental rights to data protection. The project and the experimental protocol were approved by the Ethical Board of the University of Helsinki as well as by the Ethical Board of the Universidad Loyola Andalucía. Before the study was conducted, students’ parents were informed about the evaluation and permission for their children to participate the study was sought. Parental written informed consent was obtained from all students participating in this study; no student participated without their parents’ informed consent.

Students from all the schools responded to the questionnaire. Questionnaires were implemented by the online platform *Survey Gizmo*^1^ and administered during regular school hours, in groups of 10–15 students. The student’s teachers and a member of the project team were in the classroom supervising the data collection during the students’ evaluation. The study was conducted in school computer labs or in the classroom with personal tablets. Only one school had to implement the study in pencil and paper format because of technical problems. Before the study started, students had to fill out an online consent form as well. Students were guaranteed anonymity and confidentiality and it was confirmed that participation was voluntary.

After the assessment, teachers participated in the training focused on the use of the *toolkit* and started the intervention period. Next, steps consisted on working with the *toolkit* during regular sessions for a period of 5 months. Monitoring sessions were planned in order to respond to schools’ queries and difficulties during the process.

### Analyses

First, the psychometric properties of the scales were assessed by computing the reliability trough Cronbach’s Alpha and McDonald’s omega. The values of correlations between items and total scores were explored to identify items working worst in Spanish students.

Total scores were later computed for each scale, taking into consideration that total scores should always express the same type of value; higher values indicated “more positive” results in terms of social and emotional competences and vice versa.

Second, descriptive analyses focused on the distribution of the data by extracting means standard deviations, skewness and kurtosis. After that, total scores were compared by age and gender groups in order to identify differences in scales scores connected to the characteristics of the students. Regarding the age variable, the sample was divided into two groups by following the criterion based on the school level: students in primary school (from 9 to 11 years old) and students in secondary school (from 12 to 16 years old). As previous literature does not provide conclusive results about the role of age and gender in terms of SEL, these analyses pretend to learn about dimensions which should be further developed in different groups, if differences exist. Means between groups were compared by Student *t*-test. Cohen’s *d* was computed to quantify the effect size of the differences (Cohen, 1988).

Next step consisted on calculating correlations between scales and dimensions measures with the aim of knowing the relationships between variables. To identify connections between variables will help to organize priorities when designing training programs and will provide a picture of potential interactions between dimensions.

Finally, the results obtained were compared with competences and skills included in the *toolkit* for evaluating the overlap between them and potential needs of Spanish students.

## Results

### Descriptive Statistics and Scales Reliability

In terms of reliability, all the scales were working adequately as Cronbach’s Alpha and McDonald’s omega values were higher than 0.7 (see Table 5).

**TABLE 5:** Descriptive analyses and reliability for individual scales.

**Scales**	**Range of the total scores**	**Mean**	**SD**	**Skewness**	**Kurtosis**	**Cronbach’s α**	**Omega**
SECQ- Self-awareness	5–30	25.16	3.99	−1.42	3.82	0.723	0.824
SECQ- Social awareness	5–30	21.24	5.11	−0.60	0.42	0.764	0.842
SECQ- Self-management	5–30	20.93	5.55	−0.47	−0.16	0.806	0.868
SECQ- Relationship management	5–30	24.75	4.34	−1.07	1.82	0.727	0.830
SECQ- Responsible decision-making	5–30	23.98	4.84	−1.04	1.50	0.823	0.877
Self-Esteem	5–35	25.13	5.80	−0.96	1.39	0.603	0.756
Bullying	2–10	2.43	1.03	2.92	10.03	^∗^	^∗^
Life satisfaction	1–10	8.37	1.87	−1.67	3.18	^∗^	^∗^
Teacher support	3–15	12.27	2.78	−1.10	0.88	0.815	0.890
School difficulties	3–15	5.19	3.12	1.67	2.11	0.814	0.889

*^∗^Cronbach’s alpha and omega indexes were not calculated for scales measuring bullying and life satisfaction as both had less than three items (minimum required to compute that analysis).*

Only in the self-esteem scale values were lower than the established criteria. Looking at correlations between items and total scores (Discrimination index) it was observed that item 4 was working wrongly as it was not capturing the same meaning as the rest of the scale. Looking at the item stem “I wish I could respect myself more” we concluded that the meaning in Spanish could be confusing as “respect myself” in that item could have a negative or a positive meaning and, then, students could be responding by having in mind different meanings.

Then, descriptive analyses were conducted in order to learn about the distribution of responses from Spanish students. Table 5 describes mean and standard deviation (SD) for each scale including a single-item scale as well as a range of total scores according to values assigned to response alternatives.

As Table 5 shows, means for variables indicating social and emotional competences were always above the middle point of the scale indicating a tendency to the positive pole. However, variables measuring an absence of social and emotional competences reached means lower than the middle point of the scale and tending to the negative pole. Specifically, in the SEC scale, *self-management* and *social awareness* dimensions exhibited the lowest values whereas *self-awareness* and *relationship management* reached the highest means. Looking at responses for scales measuring *bullying* and *school difficulties*, we observed that the 88.4% and the 41.6% of the students reached total scores equal or lower to 3 points respectively, whereas only 0.9% of the participants scored more than 6 points in the *bullying* scale and 5.7% scored higher than 12 points for *school difficulties.* On the other hand, 46.1% of the students scored 27 points or more in *self-esteem*, 54% scored 12 points or more in *teacher support* and 58.5% scored 9 points or more in *life satisfaction*. Skewness and kurtosis values indicate that all the scales reach acceptable ranges for univariate normality although kurtosis values for *bullying* indicate that the scale has a peaked distribution.

### Differences Between Groups

The means between age groups were compared by the Student’s *t*-test. Significant differences were found for the *self-awareness* dimension (*t* = 4.30; *p* < 0.01; *d* = 0.26) in the SECQ, *self-esteem* (*t* = 5.73; *p* < 0.01; *d* = 0.34), *bullying* (*t* = 5.72; *p* < 0.01; *d* = 0.35), *teacher support* (*t* = 15.48; *p* < 0.01; *d* = 0.93), and *school difficulties* (*t* = 1.23; *p* < 0.01; *d* = 0.07). In all the cases, younger students (7–11 years old) got higher values but according to Cohen’s *d* values, the effect size was large only for differences in the dimension of *teacher support.*

Means were compared between gender groups by the Student’s *t*-test as well. Significant differences were found for *bullying* (*t* = 3.11; *p* = 0.02; *d* = 0.16), *teacher support* (*t* = 4.04; *p* < 0.01; *d* = 0.07) and *school difficulties* (*t* = 2.17; *p* = 0.03; *d* = 0.13). Girls obtained higher mean values in *teacher support* and lower values in *bullying* and *school difficulties* but the size of the difference was small in all the cases.

### Relationships Between Scales and Dimensions

Correlations between variables were computed to check relationships between variables and dimensions (see Table 6).

**TABLE 6:** Correlations between scales.

**Scales**	**1**	**2**	**3**	**4**	**5**	**6**	**7**	**8**	**9**	**10**
(1) SECQ- Self-awareness	1									
(2) SECQ- Social awareness	0.462^∗∗^	1								
(3) SECQ- Self-management	0.464^∗∗^	0.335^∗∗^	1							
(4) SECQ- Relationship management	0.437^∗∗^	0.346^∗∗^	0.444^∗∗^							
(5) SECQ- Responsible decision-making	0.549^∗∗^	0.374^∗∗^	0.548^∗∗^	0.520^∗∗^	1					
(6) Self-Esteem	0.405^∗∗^	0.204^∗∗^	0.352^∗∗^	0.270^∗∗^	0.353^∗∗^	1				
(7) Bullying	−0.058^∗^	–0.006	–0.020	–0.099^∗∗^	–0.099^∗∗^	–0.033	1			
(8) Life satisfaction	0.240^∗∗^	0.129^∗∗^	0.250^∗∗^	0.206^∗∗^	0.251^∗∗^	0.342^∗∗^	–0.078^∗∗^	1		
(9) Teacher support	0.304^∗∗^	0.133^∗∗^	0.247^∗∗^	0.315^∗∗^	0.326^∗∗^	0.315^∗∗^	–0.020	0.355^∗∗^	1	
(10) School difficulties	–0.085^∗∗^	–0.109^∗∗^	–0.098^∗∗^	–0.091^∗∗^	–0.115^∗∗^	–.068^∗∗^	0.204^∗∗^	–0.090^∗∗^	–0.105^∗∗^	1

*^∗^*p* < 0.05 and ^∗∗^*p* < 0.01.*

As expected, significant and positive correlations were obtained between scales indicating SEC and between scales indicating the absence of SEC, whereas significant and negative correlations between scales indicate the opposite. Specifically, all the dimensions in SECQ, where positive values expressed higher levels of SEL, got significant and positive values between them and with other positive variables such as *life satisfaction* or *teacher support*. Whereas, these dimensions and positive variables were showed negative and significant correlations with variables expressing the absence of SEL as *bullying* or *school difficulties.* On the other hand, positive and significant correlations were obtained between these two variables measuring the absence of SEL (*bullying* and *school difficulties).*

Non-significant correlations were only obtained in two cases: between *teacher support* and *bullying*, and between *bullying* and the *self-management* dimension in the SECQ.

According to the results shown in Table 5, *self-management* and *social awareness* are the two dimensions of the SECQ scale scoring lower in Spanish students and, therefore, these should be emphasized by teachers when developing activities for students.

## Discussion

The aim of the paper was twofold. On one hand, the goal was to explain the steps for creating a valid tool to be applied in schools in different countries to help develop social and emotional competences. To do that, professionals from different areas were involved in the design task, and experiences were collected in order to standardize the training for teachers and students to improve social and emotional competences. On the other hand, we aimed to illustrate the potential needs of students in Spain in order to learn about those dimensions less developed in Spanish students and to provide a useful tool for professionals in charge of promoting the training or the creation of programs focused on improving SEL in students.

Regarding the process for designing the *toolkit*, we emphasized the importance of including professionals with different approaches as they can contribute to create a realistic tool. In other words, researchers sometimes focused on scientific tools while teachers are centered on their personal experiences. Neither in isolation can create the tools able to provide evidence about their utility. Therefore, it is crucial to combine both fields and incorporate both experiences and scientific knowledge for developing standard and useful procedures that can be evaluated by robust assessment tools. However, that task is not as easy as the academic field and the realities in the classrooms can walk in different directions. But, due to the benefits gathered when including different agents working on educational issues we encourage researchers to ask professionals working directly with students when designing training programs. In that way activities and procedures created will focus on main needs at the same time that they respond to teachers worries and fit to the possibilities they have in their daily work.

In relation to the results obtained from the responses of Spanish students, we discovered the dimensions that are less developed in Spain. Values found for SEC dimensions indicated that *self-management* and *social awareness* were less developed and then should go first when working or training SEL in students. As scores for *self-awareness* were high, maybe indicators within that last dimension could be used as a bridge for working the others. That means as students seem to recognize their feelings and moods, the training can start with helping to recognize these feelings and moods in others and then working with managing feeling emerging in the presence of negative situations. Therefore, this information can help strengthen teacher training to focus on those dimensions. Specifically, our results showed a negative relation between social and emotional competences and bullying habits. These findings are consistent with previous studies (see Yubero et al., 2017), hence proving the importance of social and emotional training to prevent bullying and harassment in the schools. With regard to age differences, important recent studies have indicated that the subjective well-being of adolescents decreases significantly after 12 years (see Tomyn and Cummins, 2011; Casas et al., 2012; González-Carrasco et al., 2016). Furthermore, previous findings showed a decrease in lower levels self-esteem in this age group, explained by the contextual changes experienced when students move from primary to secondary grades (see Wigfield et al., 1991). Regarding the gender differences, this results support previous studies that shows that boys are more likely to be involved in bullying (see Cook et al., 2010). However, values of effect size measures indicate that the size of these differences is small in most of the cases and, therefore, results should be taken as a hint for understanding trends.

Therefore, these findings should be considered in the *toolkit* teacher training, wherein secondary teachers are made aware of this reduction in competences in adolescent students and can focus on how the toolkit can help to increase these competences.

Some limitations of the study should be drawn. On one hand, results from Spanish students cannot be compared yet to responses from students in other countries. Next studies will focus on analyzing needs from students in other countries and on finding common elements from SEL across countries and potential connections with the students’ achievement. Once we collect data from all participating countries, we will be able to perform a cross-country comparison of the dimensions, which will offer definitive evidence of the utility of the *toolkit* based on international parameters.

Moreover, the changes in the variables after using the *toolkit* were not collected now and, therefore, arguments about the utility of the *toolkit* cannot be supported. Nevertheless, in future researches, we will investigate the variables improving and those being maintained or decreasing. That information will provide evidence to support the utility of the toolkit.

To summarize, this paper presents a proposal that contributes to reduce the gap between research and educational policies, thus improving the effectiveness of social and emotional school programs. In addition, the results offer evidence that supports innovative educational methods and policy development to promote better recognition and provide tools for improving the assessment of social and emotional competences across countries. Moreover, the study collected support for keep working on improving programs focused on SEL as according to the literature and experts’ experience it has an impact on students’ daily life.

## Data Availability

The datasets generated for this study are available on request to the corresponding author.

## Ethics Statement

The studies involving human participants were reviewed and approved by Ethical Board of the University of Helsinki. Ethical Board of the Universidad Loyola Andalucía. Written informed consent to participate in this study was provided by the participants’ legal guardian/next of kin.

## Author Contributions

PA prepared the data sets used for data analyses and wrote the second phase of the study. IL-C described the theoretical framework and was in charge of the literature search. FC and IB are principal researchers of the project in the Spanish work package. FC wrote the first phase of the study. IB was in charge of the analysis of the second phase and of connecting sections. All authors participated in the creation of the *toolkit* and the data collection as described this manuscript.

## Conflict of Interest Statement

The authors declare that the research was conducted in the absence of any commercial or financial relationships that could be construed as a potential conflict of interest.

## Footnotes

1 https://www.surveygizmo.com/

## References

[B1] Albright M. I. Weissberg R. P. (2010). “School-family partnerships to promote social and emotional learning,” in *Handbook of School-Family Partnerships*, eds Christenson S. L. Reschly A. L. (New York, NY: Taylor & Francis), 246–265.

[B2] Birch S. H. Ladd G. W. (1997). The teacher–child relationship and children’s early school adjustment. *J. Sch. Psychol.* 35 61–79. 10.1016/s0022-4405(96)00029-5

[B3] Bisquerra R. (2016). *10 ideas clave. Educación emocional*. Barcelona: Graó

[B4] Cahill H. Kern M. L. Dadvand B. Cruickshank E. W. Midford R. Smith C. (2019). An integrative approach to evaluating the implementation of social and emotional learning and gender-based violence prevention education. *Int. J. Emot. Educ.* 11 135–152.

[B5] Cantril H. (1965). *The Pattern of Human Concerns.* New Brunswick, NJ: Rutgers University Press.

[B6] Casas F. Sarriera J. C. Alfaro J. González M. Malo S. Bertran I. (2012). Testing the personal wellbeing index on 12–16 year-old adolescents in 3 different countries with 2 new items. *Soc. Indic. Res.* 105 461–482. 10.1007/s11205-011-9781-1

[B7] Casel. (2015). *CASEL Guide: Effective Social and Emotional Learning Programs: Middle and High School Edition.* Chicago, IL: Collaborative for Academic, Social, and Emotional Learning.

[B8] Catalano R. F. Fagan A. A. Gavin L. E. Greenberg M. T. Irwin C. E. Jr. Ross D. A. (2012). Worldwide application of prevention science in adolescent health. *Lancet* 379 1653–1664. 10.1016/S0140-6736(12)60238-4 22538180 PMC4398056

[B9] Cohen J. (1988). *Statistical Power Analysis for The Behavioral Sciences (2. Auflage).* Hillsdale, NJ: Erlbaum.

[B10] Collaborative for Academic, Social and Emotional Learning [CASEL] (2003). *Safe and Sound: An Educational Leader’s Guide to Evidence-Based Social and Emotional Learning (SEL) Programs.* Chicago: CASEL.

[B11] Cook C. Williams K. Guerra N. Kim T. Sadek S. (2010). Predictors of bullying and victimization in childhood and adolescence: a meta-analytic investigation. *Sch. Psychol. Q.* 25 65–83. 10.1037/a0020149

[B12] Corcoran R. P. Cheung A. C. K. Kim E. Xie C. (2018). Effective universal school-based social and emotional learning programs for improving academic achievement: a systematic review and meta-analysis of 50 years of research. *Educ. Res. Rev.* 25 56–72. 10.1016/j.edurev.2017.12.001

[B13] Currie C. Inchley J. Molcho M. Lenzi M. Veselska Z. Wild F. (2014). *Health Behaviour in School-aged Children (HBSC) Study Protocol: Background, Methodology and Mandatory Items for the 2013/14 Survey.* St Andrews, KY: CAHRU.

[B14] Devaney E. O’Brien M. U. Resnik H. Keister S. Weissberg R. P. (2006). *Sustainable Schoolwide Social and Emotional Learning (SEL): Implementation Guide and Toolkit.* Chicago, IL: CASEL, University of Illinois at Chicago.

[B15] Durlak J. A. Weissberg R. P. Dymnicki A. B. Taylor R. D. (2011). The impact of enhancing students’ social and emotional learning: a meta-analysis of school-based universal interventions. *Child Dev.* 82 405–432. 10.1111/j.1467-8624.2010.01564.x 21291449

[B16] Durlak J. A. Weissberg R. P. Pachan M. (2010). A meta-analysis of after-school programs that seek to promote personal and social skills in children and adolescents. *Am. J. Commun. Psychol.* 45 294–309. 10.1007/s10464-010-93000-6 20300825

[B17] Elliott D. S. Mihalic S. F. (2004). Issues in disseminating and replicating effective prevention programs: blending prevention research and practice in schools. *Prev. Sci.* 5 47–53. 10.1023/B:PREV.0000013981.28071.52 15058912

[B34] González-Carrasco M. Casas F. Malo S. Viñas F. Dinisman T. (2017). Changes with age in subjective well-being through the adolescent years: differences by gender. *J. Happiness Stud*. 18, 63–88. 10.1007/s10902-016-9717-1

[B18] Greenberg M. T. Weissberg R. P. O’Brien M. U. Zins J. E. Fredricks L. Resnik H. (2003). Enhancing school-based prevention and youth development through coordinated social, emotional, and academic learning. *Am. Psychol.* 58 466–474. 10.1037/0003-066X.58.6-7.466 12971193

[B19] Harkness J. Schoua-Glusberg A. (1998). “Questionnaires in translation,” in *Cross-Cultural Survey Equivalence*, ed. Harkness J. (Mannheim: ZUMAz), 87–126.

[B20] Oberle E. Domitrovich C. E. Meyers D. C. Weissberg R. P. (2016). Establishing systemic social and emotional learning approaches in schools: a framework for schoolwide implementation. *Camb. J. Educ.* 46 277–297. 10.1080/0305764X.2015.1125450

[B21] OECD. (2015). *Skills for Social Progress: The Power of Social and Emotional Skills, OECD Skills Studies.* Paris: OECD Publishing.

[B22] Olweus D. (1996). *The Revised Olweus Bully/Victim Questionnaire Research Center for Health Promotion (HEMIL Center).* Bergen: University of Bergen.

[B23] Rimm-Kaufman S. E. Hamre B. K. (2010). The role of psychological and developmental science in efforts to improve teacher quality. *Teach. Coll. Record* 112 2988–3023.

[B24] Rosenberg M. (1979). *Rosenberg Self-esteem Scale.* New York, NY: Basic Books.

[B25] Taylor R. D. Oberle E. Durlak J. A. Weissberg R. P. (2017). Promoting positive youth development through school-based social and emotional learning interventions: a meta-analysis of Follow-Up effects. *Child Dev.* 88 1156–1171. 10.1111/cdev.12864 28685826

[B26] Tomyn A. J. Cummins R. A. (2011). The subjective wellbeing of high-school students: validating the personal wellbeing index—school children. *Soc. Indic. Res.* 101 405–418. 10.1007/s11205-010-9668-6

[B27] Tuominen-Soini H. Salmela-Aro K. Niemivirta M. (2008). Achievement goal orientations and subjective well-being: a person-centred analysis. *Learn. Instr.* 18 251–266. 10.1016/j.learninstruc.2007.05.003

[B28] Wigfield A. Eccles J. S. Mac Iver D. Reuman D. A. Midgley C. (1991). Transitions during early adolescence: changes in children’s domain-specific self-perceptions and general self-esteem across the transition to junior high school. *Dev. Psychol.* 27 552 10.1037//0012-1649.27.4.552

[B29] Yang W. P. Datu J. A. D. Lin X. Y. Lau M. M. Li H. (2019). Can early childhood curriculum enhance social-emotional competence in low-income children? A meta-analysis of the educational effects. *Early Educ. Dev.* 30 36–59. 10.1080/10409289.2018.1539557

[B30] Yubero S. Navarro R. Elche M. Larrañaga E. Ovejero A. (2017). Cyberbullying victimization in higher education: an exploratory analysis of its association with social and emotional factors among Spanish students. *Compu. Hum. Behav.* 75 439–449. 10.1016/j.chb.2017.05.037

[B31] Zhou M. Ee J. (2012). Development and validation of the social emotional competence questionnaire (SECQ). *Int. J. Emot. Educ.* 2 27–42.

[B32] Zins J. E. Elias M. J. (2007). Social and emotional learning: promoting the development of all students. *J. Educ. Psychol. Consult.* 17 233–255. 10.1080/10474410701413152

[B33] Zins J. E. Weissberg R. P. Wang M. C. Walberg H. J. (2004). *Building Academic Success Through Social and Emotional Learning: What Does the Research Say?* New York, NY: Teachers College Press.

